# PCI Deferral Based on Fractional Flow Reserve or Optical Coherence Tomography: Two-Year Results of the Forza Trial

**DOI:** 10.31083/j.rcm2311361

**Published:** 2022-10-25

**Authors:** Antonio Maria Leone, Francesco Burzotta, Cristina Aurigemma, Aniello Zambrano, Giuseppe Zimbardo, Domenico Galante, Federico Di Giusto, Francesco Bianchini, Rocco Vergallo, Carlo Trani, Filippo Crea

**Affiliations:** ^1^Dipartimento di Scienze Cardiovascolari, Fondazione Policlinico Universitario A, Gemelli IRCCS, 00168 Roma, Italy; ^2^Dipartimento di Scienze Cardiovascolari , Università Cattolica del Sacro Cuore, 00168 Roma, Italy; ^3^Cardiologia e Unità terapia intensiva, Policlinico Casilino, 00169 Roma, Italy

**Keywords:** fractional flow reserve, optical coherence tomography, FFR, OCT, personalized medicine

## Abstract

**Backgroud:**

The “FFR or OCT Guidance to Revascularize Intermediate Coronary Stenosis Using Angioplasty” (FORZA) trial showed that in patients with angiographically intermediate coronary lesions (AICLs), optical coherence tomography (OCT) guidance of percutaneous coronary intervention (PCI) reduced the occurrence of the composite endpoint of major adverse cardiac events (MACE) or significant angina at 13 months, while fractional flow reserve (FFR) guidance was associated with a higher rate of medical management and with lower costs. Safety of PCI deferral when FFR >0.80 is known, while data on clinical outcomes using an OCT guidance are lacking. We assessed the safety of PCI deferral based on OCT findings.

**Methods:**

This is a subgroups analysis of the FORZA Trial focusing on the clinical outcome of patients in whom PCI was originally deferred. In details, patients with AICLs were randomized to FFR or OCT imaging. In the FFR arm, PCI was deferred if FFR was >0.80 while in the OCT arm in the absence of any of the following conditions: area stenosis >75%, or 50% to 75% with minimum lumen area <2.5 ${\mathrm{mm}}^{2}$ or plaque rupture. Angina status (evaluated using the Seattle Angina Questionnaire, SAQ), MACE (death, myocardial infarction, target vessel revascularization) and rate of patients treated with optimal medical therapy alone were assessed at 24 months.

**Results:**

From a total of 350 patients with 446 AICLs enrolled in the trial (176 randomized to FFR and 174 to OCT), based on the predefined FFR and OCT criteria, PCI was deferred in 119 patients (67.6%) in the FFR arm, and in 82 patients (47.1%) in the OCT arm. At 24-months follow-up, significant residual angina (defined as a value <90 on the angina frequency scale) was observed in 6 patients (5.0%) in the FFR arm, and in 6 patients (7.3%) in the OCT arm (*p *= 0.55). Rate of MACE was 10.9% in the FFR arm and 6.1% in the OCT arm (*p *= 0.32). The number of patients managed by optimal medical therapy alone was still significantly higher using FFR than OCT guidance also at 24 months (60.2% vs 44.2%, *p *= 0.0038).

**Conclusions:**

PCI-deferral based on OCT (using the FORZA trial criteria) is safe and associated with numerically less events at 24-months follow up. FFR guidance is still associated with a higher number of patients managed by optimal medical therapy alone.

## 1. Introduction

Functional assessment of intermediate coronary stenoses by means of fractional flow reserve (FFR) has proven to be better than angiography alone in selecting lesions to be treated and in guiding percutaneous coronary intervention (PCI) [1, 2, 3]. In contrast, intracoronary imaging techniques, such as optical coherence tomography (OCT), despite useful in optimizing PCI, does not still play a clear role when it comes to choosing the lesions to treat [4, 5, 6]. The open-label, single-centre, prospective, randomized “FFR or OCT Guidance to Revascularize Intermediate Coronary Stenosis Using Angioplasty” (FORZA) trial [7] (NCT01824030) was therefore conducted in order to compare the clinical and economic implications of PCI-deferral of angiographically intermediate coronary lesions (AICLs) based on OCT evaluation or on FFR assessment. In the present report, the 24-months follow-up results of the subgroup of patients in which a strategy of FFR or OCT guided strategy of PCI deferral of the FORZA trial are presented.

## 2. Methods

### 2.1 Study Design 

FORZA trial [7] enrolled three hundred and fifty consecutive patients with stable ischemic heart disease or stabilized (culprit lesion treated previously) acute coronary syndrome and evidence of at least one AICL, for a total of 446 AICL. AICL was defined as a coronary lesion with an angiographically estimated percentage diameter stenosis ranging from 30% and 80%.

**Fig. 1.**
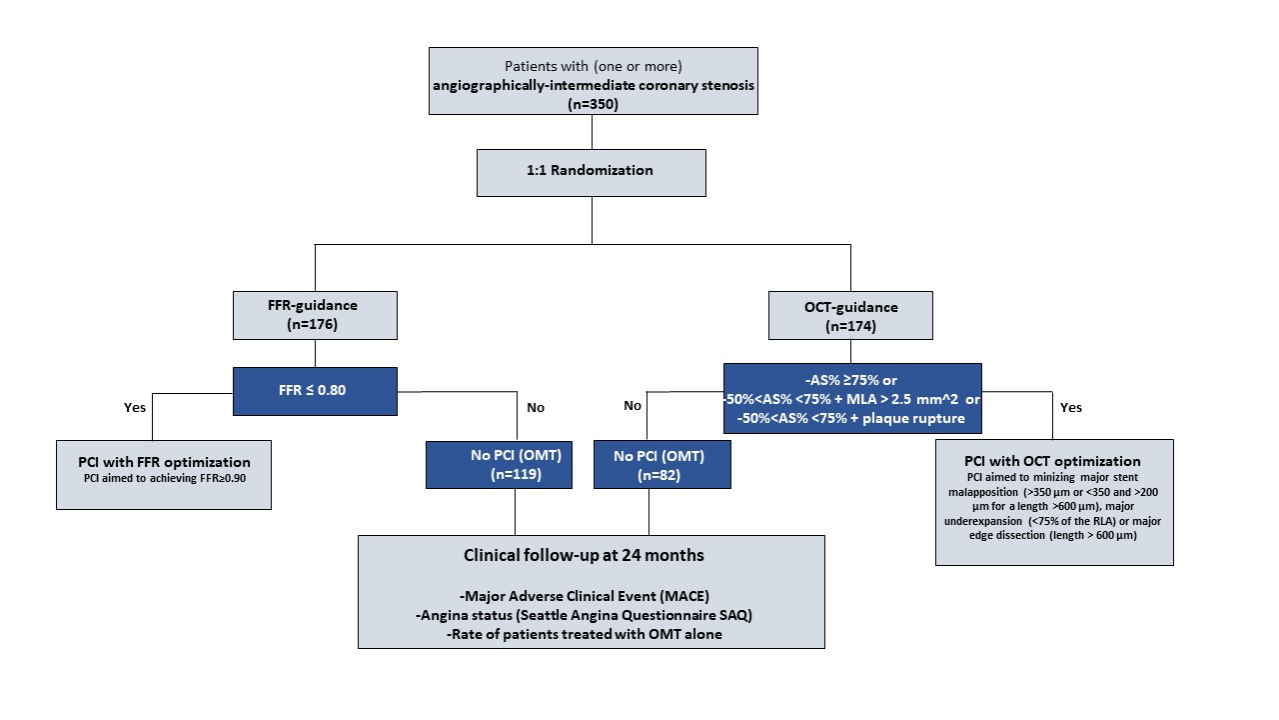
**Study flow chart**. AS, area stenosis; FFR, fractional flow reserve; MLA, minimal luminal area; OCT, optical coherence tomography; PCI, percutaneous coronary intervention; OMT, optical medical therapy.

Patients were randomized in a 1:1 fashion to the use of OCT guidance or FFR guidance for deferring or performing PCI. Specific inclusion and exclusion criteria were previously reported [7]. The study was approved by the ethics committee of our institution (internal code 6261/13), and all patients signed a dedicated informed consent form. The data that support the findings of this study are available from the corresponding author upon reasonable request. We have already published results at 1 and 13 months. We selected patients in whom PCI, based on FFR and OCT criteria, was initially deferred. In details, PCI was deferred when FFR was >0.80 or, in the OCT arm, in the absence of any of the following conditions: an area stenosis >75%, or 50% to 75% with minimum lumen area <2.5 ${\mathrm{mm}}^{2}$ or plaque rupture. The study flowchart is summarized in Fig. 1.

### 2.2 Procedure Description 

After placement of a guiding catheter at the coronary ostium, FFR or OCT assessments have been performed according to randomization as described previously [7]. Randomization was based on a computer-generated random series of numbers and took place through the opening of an envelope in which the treatment arm was reported. Both the operator and the patient were unblinded to the technique used.

### 2.3 Definition of Deferred Groups 

#### 2.3.1 FFR Deferred Arm

**Fig. 2.**
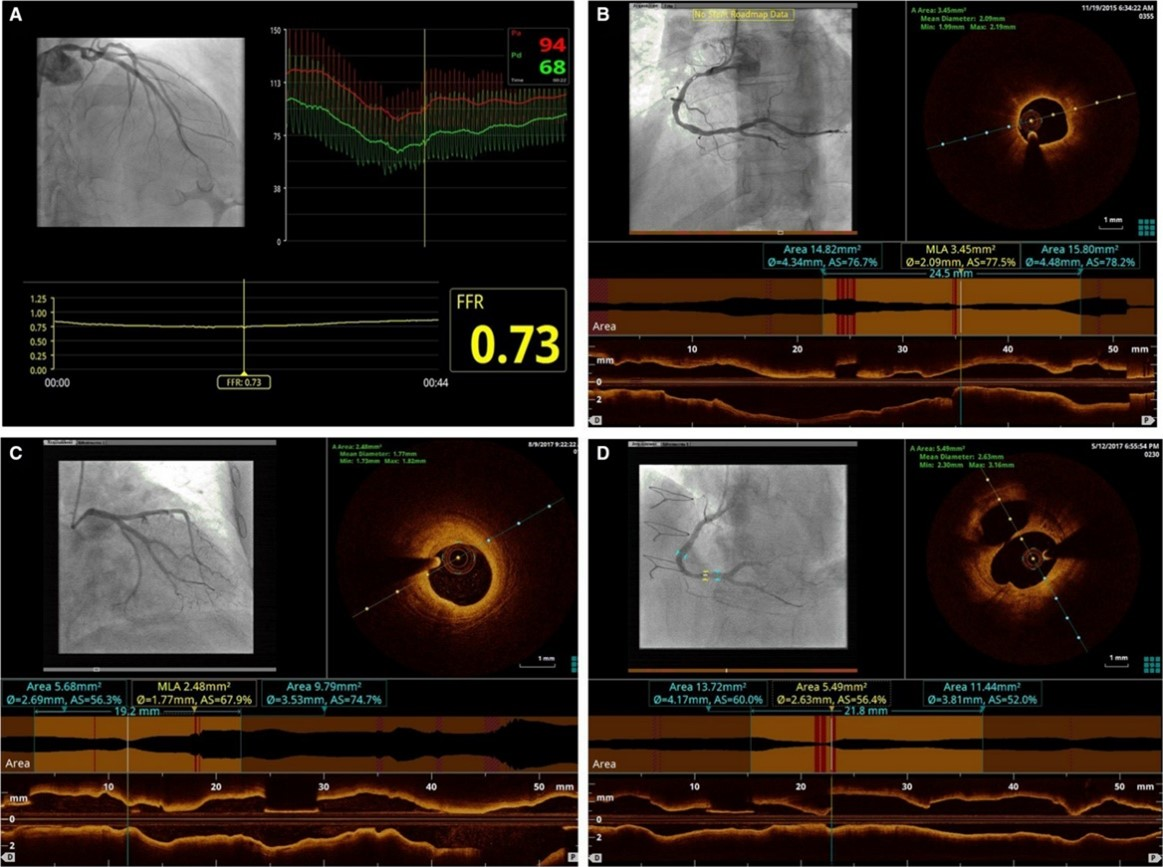
**The FORZA criteria for AICLs revascularization based on FFR or OCT**. (A) FFR ≤0.80. (B) AS% ≥75%. (C) AS% from 50% to 75% with MLA <2.5 ${\mathrm{mm}}^{2}$. (D) AS% from 50% to 75% and plaque ulceration. AICLs, angiographically intermediate coronary lesions; FFR, fractional flow reserve; OCT, optical coherence tomography; AS, area stenosis; MLA, minimal lumen area.

A 0.014-inch pressure-monitoring guidewire (Pressure Wire Certus or Aeris; Abbott Vascular, Abbott Park, IL, USA) was advanced beyond the AICL under radioscopic examination to calculate the lowest ratio of distal coronary pressure (Pd) divided by aortic pressure (Pa) after achievement of hyperaemia using adenosine. Lesions were deferred when the FFR values was >0.80. In contrast, when FFR was ≤0.80, PCI was performed with the aim of achieving a post-stenting FFR ≥0.90 (Fig. 2).

#### 2.3.2 OCT Deferred Arm

OCT images were acquired (after intracoronary administration of nitro-glycerine) at the site of the AICL with commercially available systems (C7 System, LightLab Imaging/St. Jude Medical, Westford, Massachusetts; and, after its availability, Optis System, Abbott Vascular) after the OCT catheter (C7 Dragonfly, LightLab Imaging/St. Jude Medical; and Dragonfly Optis, Abbott Vascular) was advanced to the distal end of the target lesion. The entire length of the region of interest was evaluated: minimal luminal area (MLA) (defined as cross-sectional area at the smallest luminal area level), proximal reference luminal area (RLA) (defined as the cross section at the frame with largest lumen within 10 mm proximal to MLA and before any major side branch), distal RLA (defined as the cross section at the frame with largest lumen within 10 mm distal to MLA and before any major side branch), and mean RLA (defined as [proximal RLA + distal RLA]/2). On the basis of these parameters, percentage of area stenosis (AS) was calculated using the following formula: (mean RLA - MLA) / mean RLA × 100. Plaque rupture (also called ulceration) was defined as a recess in the plaque beginning at the luminal-intimal border [8].

PCI was deferred in the absence of any of the following conditions: (1) area stenosis >75%; (2) area stenosis between 50% and 75% with minimum lumen area <2.5 ${\mathrm{mm}}^{2}$; (3) plaque rupture (Fig. 2).

If at least 1 of the previous criteria occurred, operator proceeded to PCI. In patients who underwent revascularization, OCT was used also to guide and optimize PCI results. Optimization was performed in the presence of major stent malapposition (defined as distance between strut and vessel wall >350 μm or <350 μm and >200 μm for a length >600 μm), major underexpansion (in-stent minimal cross-sectional area <75% of the RLA) or major edge dissection (defined as length >600 μm).

### 2.4 Study Endpoints

The study evaluates the clinical end point of “angina status” using the significant residual angina (<90 score at SAQ angina frequency scale) plus major adverse cardiovascular events (MACE), defined as the occurrence of death, spontaneous myocardial infarction (MI), and target vessel revascularization at 24 months. The prevalence of the individual components of the primary combined endpoint were considered secondary endpoints. The full description of each clinical outcomes is described in the main paper [7].

Other outcomes of special interest were the SAQ value and the variation of angina symptoms after the 2 years of follow-up as well the overall number of medically managed patients. In addition, radioscopic time (min), amount of contrast medium (mL), post procedural length of hospitalization (days) and estimated costs associated with the two different strategies were evaluated as further secondary endpoints.

The total costs, including the cost of the first and any unplanned hospitalization after discharge was evaluated and compared between arms. Details for procedural and hospitalization cost evaluation have been previously reported [7].

This sub-study was designed to test the hypothesis that OCT imaging could be an acceptable alternative to FFR for deferral patients with AICLs after 24 months of follow-up.

### 2.5 Statistical Analysis

Categorical variables were expressed as percentages and analyzed by Fisher’s exact test. Continuous variables (including clinical and economical end points) are expressed as mean ± SD and/or median [interquartile range] and compared using the paired and unpaired *t*-test or the nonparametric Wilcoxon and Mann–Whitney U-test, as appropriate, after having tested normality using the Kolmogorov–Smirnov test. Differences were considered significant with *p *< 0.05. Missing values were not counted, and all analysis were based only on valid values and performed by intention to treat using GraphPad Prism (version 5.0; GraphPad Software Inc., San Diego, CA, USA) and SPSS software (v.21.0; SPSS, Inc., Chicago, IL, USA).

## 3. Results

The characteristics of the patients and lesions enrolled in the two study arms are showed in Tables 1,2 [8]. In the FFR group, PCI was deferred in 119 patients (67.6%) vs 82 (47.1%) in the OCT group. Deferred patients according to FFR or OCT were fairly similar (no statistically significant difference in baseline clinical characteristic except for a significant higher statin and oral anticoagulation use in patients evaluated by OCT). In the OCT group, higher prevalence of multivessel disease and LCX involvement were noted. Mean FFR was 0.87 ± 0.05 in deferred FFR group while mean minimal lumen area was 3.74 ± 1.94 ${\mathrm{mm}}^{2}$ and area stenosis 53 ± 18% in the deferred OCT group.

**Table 1.** **Patients clinical characteristics**.

		All patients	FFR	OCT	*p* value
		n = 201	n = 119	n = 82	
Age		68 ± 9	70 ± 10	68 ± 9	0.41
Male gender		89 (74.6%)	80 (67.2%)	66 (55.5%)	0.22
BMI		27 ± 4	27 ± 4	27 ± 4	0.5
Risk factors					
	Diabetes	67 (33%)	39 (32.8%)	28 (34.1%)	0.88
	Hypertension	170 (84.6%)	102 (85.7%)	68 (82.9%)	0.69
	Dyslipidemia	141 (70.1%)	81 (68.1%)	60 (73.2%)	0.54
	Smoking	73 (36.3%)	47 (39.5%)	26 (31.7%)	0.24
	CKD	40 (19.9%)	26 (21.8%)	14 (17.1%)	0.47
Previous history					
	Previous PCI	83 (41.3%)	51 (42.9%)	32 (39%)	0.27
	Previous CABG	4 (2.0%)	2 (1.73%)	2 (2.4%)	1
	Previous MI	44 (21.9%)	20 (16.8%)	24 (29.3%)	0.06
Clinical presentation					
	Stable ischemic heart disease	158 (78.6%)	94 (79%)	64 (78.0)	0.86
	ACS	43 (21.4%)	25 (21%)	18 (21.9%)	0.68
	Unstable angina	28 (13.9%)	16 (13.4%)	12 (14.6%)	0.84
	NSTEMI	14 (7.0%)	9 (7.6%)	5 (6.1%)	0.78
	STEMI	1 (0.5%)	0 (0%)	1 (1.2%)	0.41
	LVEF (%)	57 ± 8	57 ± 7	58 ± 9	0.55
	Seattle Angina Questionnaire	83 ± 22	84 ± 21	85 ± 22	0.62
Therapy at discharge					
	Aspirin	165 (82.1%)	97 (81.5%)	68 (82.9%)	0.24
	P2Y12 inhibitors	87 (43.3%)	48 (40.3%)	39 (47.6%)	0.31
	Beta blockers	145 (72.1%)	80 (67.2%)	65 (79.3%)	0.65
	Calcium channel blockers	51 (25.4%)	27 (22.7%)	24 (29.3%)	0.63
	ACE inhibitors/ARB	145 (72.1%)	86 (72.3%)	59 (71.9%)	1
	Statin	162 (80.6%)	89 (74.8%)	73 (89.0%)	0.02
	Nitrates	14 (7.0%)	7 (5.9%)	7 (8.5%)	0.78
	Ranolazine	16 (8.0%)	9 (7.6%)	7 (8.5%)	1
	Diuretics	56 (27.9%)	32 (26.9%)	24 (29.3%)	1
	Oral anticoagulant	24 (11.9%)	9 (7.6%)	15 (18.3%)	0.03

FFR, fractional flow reserve; OCT, optical coherence tomography; BMI, body mass index; CKD, chronic kidney disease; PCI, percutaneous coronary intervention; CABG, coronary artery bypass grafting; MI, myocardial infarction; ACS, acute coronary syndromes; NSTEMI, non ST elevation myocardial infarction; STEMI, ST elevation myocardial infarction; LVEF, left ventricular ejection fraction; ACE, Angiotensin-converting enzyme; ARB, angiotensin receptor blocker.

**Table 2.** **Lesions characteristics**.

		FFR	OCT	*p *value
		151 lesions	102 lesions	
Multivessel disease		27 (22.7%)	32 (39%)	0.018
Studied lesions		151	102	1
Single lesion studied		87 (57.6%)	66 (64.7%)	0.36
>1 lesion studied		64 (42.4%)	36 (35.3%)	
Target lesion				
	LAD	93 (%)	64 (%)	
	LCX	25 (%)	10 (%)	0.02
	RCA	28 (%)	20 (%)	
	Visual diameter stenosis (%)	51 ± 8	52 ± 8	0.19
Baseline findings according to technique of randomization				
	Resting Pd/Pa	0.95 ± 0.03	N/A	
	FFR	0.87 ± 0.05	N/A	
	MLA (${\mathrm{mm}}^{2}$)	N/A	3.74 ± 1.94	
	AS (%)	N/A	53 ± 18	

FFR, fractional flow reserve; OCT, optical coherence tomography; LAD, left anterior descending artery; LCX, left circumflex artery; RCA, right coronary artery; MLA, minimal lumen area; AS, area stenosis.

Bifurcation lesions were slightly more prevalent in the OCT arm, without significant differences in terms of prevalence and revascularization adopted strategy (single stent or double stents) (Table 3).

**Table 3.** **Bifurcation Lesions and treatment FORZA trial**.

	FFR	OCT	*p* value
	225 lesions	221 lesions	
Bifurcation PCI (%)	17 (7.5)	24 (10.9)	0.24
Single stent treatment (%)	13 (5.8)	22 (9.9)	0.31
Double stent treatment (%)	4 (1.8)	2 (0.9)	0.08

FFR, fractional flow reserve; OCT, optical coherence tomography; PCI percutaneous coronary intervention.

Radioscopic time, dose area product (DAP), consumption of contrast medium and postprocedural length of stay were numerically, but not significantly, higher in OCT than in FFR group (Table 4).

**Table 4.** **Overall procedural results**.

	FFR	OCT	*p* value
	119 patients	82 patients	
	151 lesions	102 lesions	
Radioscopic time (min)	13.1 ± 7	14.4 ± 23.6	0.58
DAP (mGy*${\mathrm{cm}}^{2}$)	16127 ± 21387	19645 ± 24891	0.29
Contrast media (mL)	185 ± 90	202 ± 99	0.21
Post procedural length of stay (days)	2.5 ± 1.5	3.0 ± 3.8	0.21

FFR, fractional flow reserve; OCT, optical coherence tomography; DAP, dose area product.

**Fig. 3.**
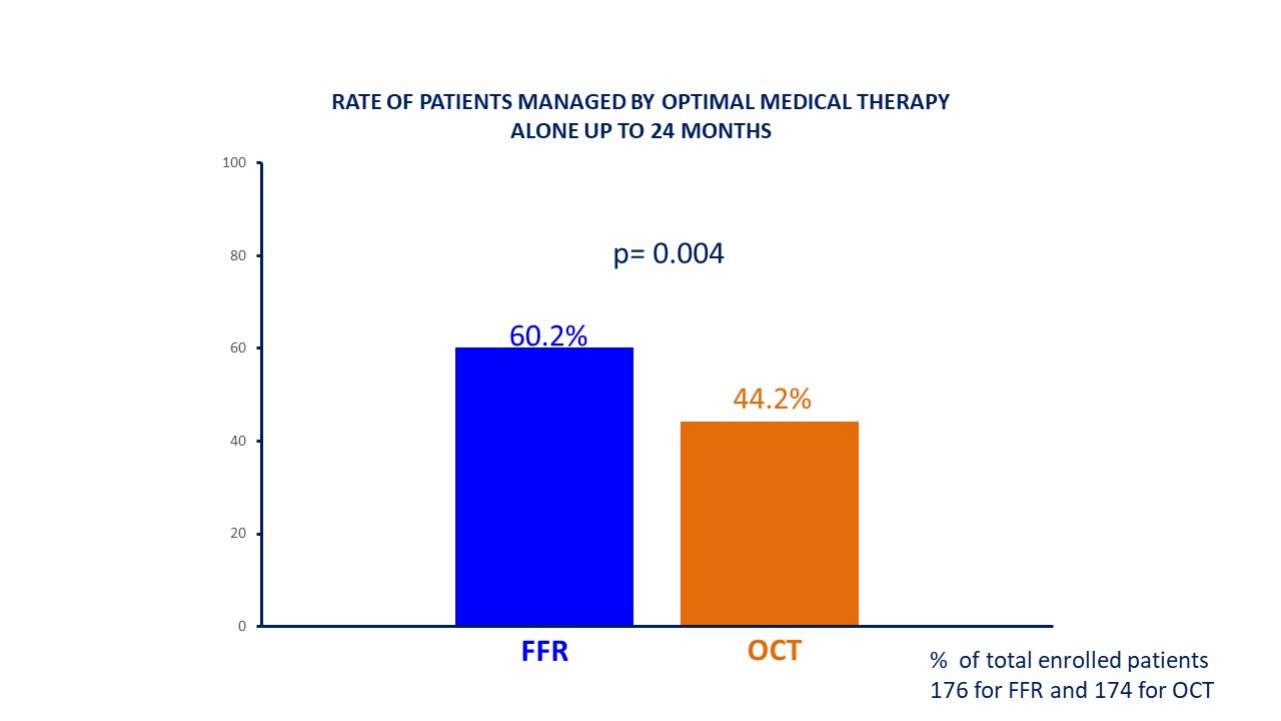
**Rate of patients managed with optimal medical therapy alone**. FFR, fractional flow reserve; OCT, optical coherence tomography.

**Fig. 4.**
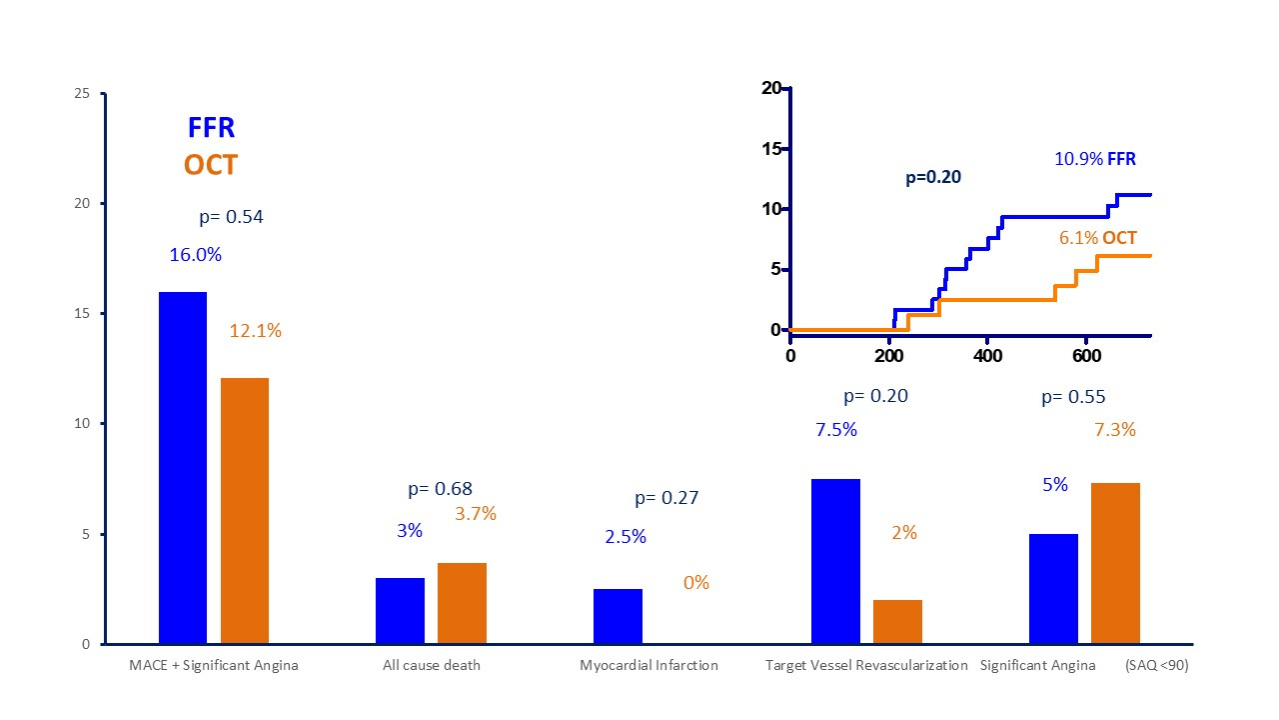
**Prevalence of different endpoints and Kaplan Meier curves for MACE**. MACE, major cardiovascular events; SAQ, Seattle Angina Questionnaire; FFR, fractional flow reserve; OCT, optical coherence tomography.

**Fig. 5.**
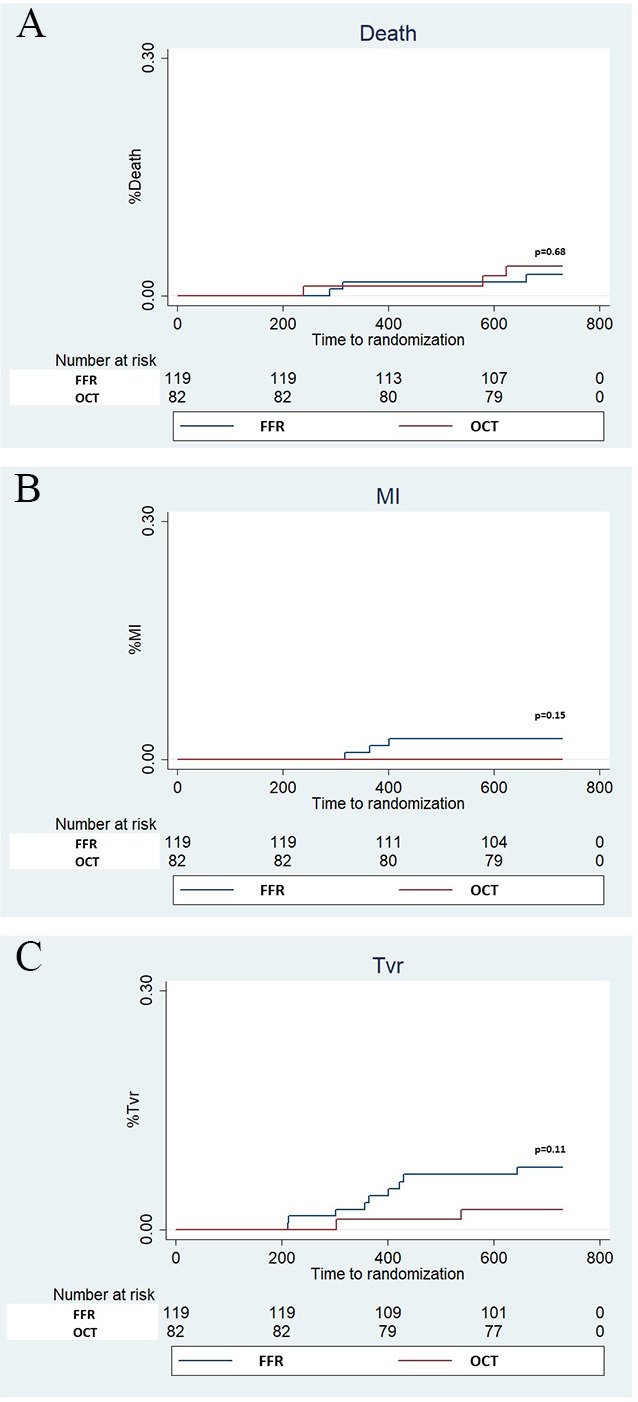
**Kaplan Meier curves for Death**, **myocardial infarction (MI) and Target vessel revascularization (TVR)**. Panel A (Death), Panel B (MI) and Panel C (TVR).

**Fig. 6.**
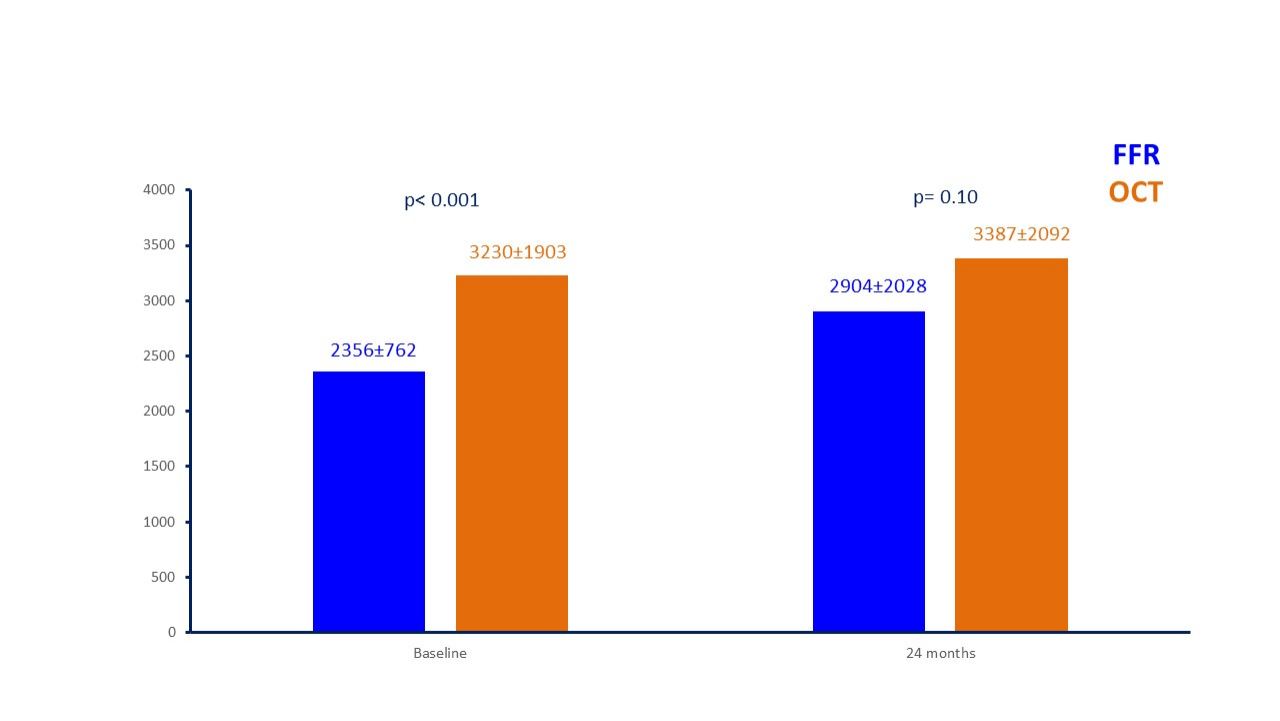
**Costs for patients managed with FFR and OCT at baseline and at 24-month follow-up (in euros)**. FFR, fractional flow reserve; OCT, optical coherence tomography.

The two groups had a similar value of frequency of angina at SAQ at enrolment and both groups improved similarly at 24 months follow-up (from 82 ± 21 to 98 ± 7 in FFR, *p *< 0.001 and from 87 ± 20 to 98 ± 5 in OCT, *p *< 0.001; delta SAQ in FFR 16 ± 20 vs delta SAQ in OCT 10 ± 22, *p* = 0.09) (Table 5). More importantly, the prevalence of persisting significant angina (defined as <90 in frequency scale of SAQ) was similar in FFR and OCT deferred patients (5% vs 7.3%, *p* = 0.55) (Table 5). Overall, after 24 months of follow up after the index procedure with eventual PCI deferral, the rate of patients managed with optimal medical therapy alone was still significantly higher in FFR group in comparison to OCT (60% vs 44.2%, *p* = 0.004) (Fig. 3). As shown in Fig. 4, 24-month MACE were numerically (albeit not statistically significant) higher in FFR arm (10.9% vs 6.1%). Not significant differences were observed in terms of individual endpoints between groups (TVR 7.5% FFR vs 2.4% OCT, *p* = 0.20, MI 2.5% FFR vs 0% OCT, *p* = 0.27, death 2.5% FFR vs 3.7% OCT, *p* = 0.68) (Figs. 4,5). Such additional events caused further hospitalizations and procedures during the 24 months follow-up so that costs (which were significantly lower at baseline with FFR) were not statistically significant different between the FFR and OCT deferred patients at 24 months (2904 ± 2028 vs 3387 ± 2092 euros, *p* = 0.10) (Fig. 6).

**Table 5.** **Overall endpoints results**.

Event		FFR (N = 119)	OCT (n = 82)	*p* value
Death		3 (2.5%)	3 (3.7%)	0.68
Myocardial infarction		3 (2.5%)	0 (0%)	0.27
Death/MI		6 (5%)	2 (2.4%)	0.48
TVR		9 (7.5)	2 (2.4%)	0.20
MACE		13 (10.9%)	5 (6.1%)	0.32
TVF		11 (9.2%)	4 (4.9%)	0.29
SAQ<90		6 (5%)	6 (7.3%)	0.55
MACE + angina		19 (16%)	10 (12.1%)	0.54
Optimal Medical Therapy (% of the starting population)				
	Baseline	119 (67.6%)	82 (47.1%)	<0.001
	13 months	110 (62.5%)	80 (46.0%)	<0.001
	24 months	106 (60.2%)	77 (44.2%)	0.0038
Cost at baseline		2356 ± 762	3230 ± 1903	<0.001
Cost at follow up		2904 ± 2028	3387 ± 2092	0.10

FFR, fractional flow reserve; OCT, optical coherence tomography; MI, myocardial infarction; TVR, target vessel revascularization; MACE, major cardiovascular events; TVF, target vessel failure; SAQ, Seattle Angina Questionnaire.

## 4. Discussion

A strategy of safe deferral of revascularization is possible only when spontaneous cardiovascular events are lower than the predicted events due to the procedure. A large body of evidence supports the safety of PCI deferral in case of FFR >0.80 [1] and current guidelines on PCI recommend the use of FFR (or instantaneous wave-free ratio) to assess the hemodynamic relevance of intermediate-grade coronary stenoses. On the contrary, data on clinical outcomes associated with deferral of PCI based on OCT findings are scarce and no standard anatomic references are described to evaluate the relevance of intermediate stenoses. Thus, we performed a novel analysis of the FORZA trial after an extended follow-up time of 24 months, comparing the subgroup of patients in which PCI was originally deferred based on FFR or OCT.

So far, we observed that FFR was associated with lower costs and higher medical management rate while OCT reduced the composite endpoint of major adverse cardiac events (MACE) or significant angina at 13-month [9, 10].

At 24-month of follow up FFR was still associated with a larger number of conservatively treated lesions translating into a larger and significantly higher number of medically managed patients. The high medical management rate and the overall low number of events confirmed FFR as the best and safe strategy to defer PCI.

On the other hand, the higher rate of performed PCI in the OCT arm was in line with the old previous studies, showing that visual assessment at the angiography [2], as well anatomical findings obtained by IVUS, were constantly associated with a higher rate of anatomically significant stenosis in comparison to functionally significant stenosis according to FFR.

Differently from what initially assumed in the main paper regarding the safety of “conservative” OCT criteria, a non-significant difference of MACE between the functional and imaging deferred arms was documented, with numerically less events in the OCT group.

In details, a similar death rate and a less rate of MI and TVR in OCT patients were detected underling the ability of imaging evaluation to characterize the natural history of coronary lesions, excluding the presence of high-risk features of untreated plaques and resulting a safe technique to defer PCI without impact on patient’s prognosis. On the other hand, the non-significant increase in TVR and hospitalizations in FFR arm affected total costs, which became not more statistically different at extended follow up of 24 months.

A similar value of frequency of angina at SAQ and, more importantly, a similar prevalence of persisting significant angina was noted between two arms. These data disclosed the ability of the “conservative” OCT criteria to be associated with hemodynamic significance of lesions and to predict stenosis-related myocardial ischemia in a way like FFR. We suggested a new role of pre-PCI OCT: not only to characterize coronary lesions and correctly select PCI materials in a “imaging-guided PCI” but also as an acceptable alternative for deferral angioplasty in intermediate lesions.

This study represents the first evidence that an anatomical guidance using OCT imaging, applying the simple FORZA criteria, could be an acceptable alternative to the physiological reference standard also for deferral of AICLs.

## 5. Future Directions

These are the first data supporting the use of OCT to safely defer treatment of AICL taking FFR as the gold standard. Despite the quite limited number of patients enrolled in this sub-analysis of the FORZA trial could limit the ability to draw definitive conclusions about safety of OCT to safely defer treatment of AICL, the numerically lower rate of event seen in comparison to FFR seems quite reassuring. We believe that an imaging evaluation of intermediate coronary lesions could be a comparable strategy to defer PCI like FFR, and at the same time we suppose a possible link between morphologic plaque assessment and hemodynamic significance of lesions in order to predict stenosis-related myocardial ischemia. We strongly support further investigations in this field, in particular more data are needed to assess the correlation between anatomical and functional indexes in order to predict MACE and myocardial ischemia.

## 6. Limitations

Our data derived from a single centre study, with a small sample size and the result should be regarded as hypotheses generating. It was conducted locally without either a structured clinical research organization or an independent clinical events committee. The use of unconventional OCT criteria has to be acknowledged in light of the lack of clear data at the time of design of the trial [7]. However, after initiation of the study, these criteria were validated in comparison to FFR in a retrospective cohort of patients assessed with both FFR and OCT [11] and a recent randomized trial showed a safe profile of the OCT criteria to defer or perform PCI [12]. FFR assessment was made in according to the best clinical practise, however a clear definition of diffuse versus focal disease was not available because of the absence of new tools (PPG index) [13] at the start of the study. However, pullback manoeuvres were performed both in the pre and post PCI phase to assess the pressure drop distribution along the vessel. Total procedural time was not recorded but we evaluated the “procedural radioscopic time” as a surrogate, less dependent by logistic or procedural confounders.

## 7. Conclusions

The 24-months follow-up results of the FORZA trial, the first prospective randomized trial comparing OCT and FFR to guide PCI decision and performance in AICLs, showed that deferral of PCI based on OCT is clinically safe as compared with the (contemporary gold-standard) FFR-guided approach. Over 2 years, FFR-guidance warranted higher rate of optimal medical therapy management alone as compared with OCT-guidance.
